# Sulfated motifs in heparan sulfate inhibit *Streptococcus pneumoniae* adhesion onto fibronectin and attenuate corneal infection

**DOI:** 10.1002/pgr2.9

**Published:** 2023-08-09

**Authors:** Atsuko Hayashida, Hajirah N. Saeed, Fuming Zhang, Yuefan Song, Jian Liu, William C. Parks, Paulo J. M. Bispo, Pyong Woo Park

**Affiliations:** 1 Department of Medicine, Boston Children’s Hospital, Boston, Massachusetts, USA; 2 Department of Ophthalmology, Boston Children’s Hospital, Boston, Massachusetts, USA; 3 Department of Ophthalmology, Massachusetts Eye and Ear, Boston, Massachusetts, USA; 4 Department of Chemical and Biological Engineering, Rensselaer Polytechnic Institute, Troy, New York, USA; 5 Division of Medicinal Chemistry, University of North Carolina, Chapel Hill, North Carolina, USA; 6 Department of Medicine, Cedars-Sinai Medical Center, Los Angeles, California, USA; 7 Department of Pediatrics, Harvard Medical School, Boston, Massachusetts, USA

**Keywords:** bacterial adhesion, corneal epithelial cell, extracellular matrix, fibronectin, heparan sulfate, heparin, *Streptococcus pneumoniae*

## Abstract

A large number of bacterial pathogens bind to host extracellular matrix (ECM) components. For example, many Gram-negative and Gram-positive pathogens express binding proteins for fibronectin (FN) on their cell surface. Mutagenesis studies of bacterial FN-binding proteins have demonstrated their importance in pathogenesis in preclinical animal models. However, means to draw on these findings to design therapeutic approaches that specifically target FN-bacteria interactions have not been successful because bacterial pathogens can elaborate several FN-binding proteins and also because FN is an essential protein and likely a nondruggable target. Here we report that select heparan compounds potently inhibit *Streptococcus pneumoniae* infection of injured corneas in mice. Using intact heparan sulfate (HS) and heparin (HP), heparinase-digested fragments of HS, HP oligosaccharides, and chemically or chemoenzymatically modified heparan compounds, we found that inhibition of *S. pneumoniae* corneal infection by heparan compounds is not mediated by simple charge effects but by a selective sulfate group. Removal of 2-*O*-sulfates significantly inhibited the ability of HP to inhibit *S. pneumoniae* corneal infection, whereas the addition of 2-*O*-sulfates to heparosan (H) significantly increased H’s ability to inhibit bacterial corneal infection. Proximity ligation assays indicated that *S. pneumoniae* attaches directly to FN fibrils in the corneal epithelial ECM and that HS and HP specifically inhibit this binding interaction in a 2-*O*-sulfate-dependent manner. These data suggest that heparan compounds containing 2-*O*-sulfate groups protect against *S. pneumoniae* corneal infection by inhibiting bacterial attachment to FN fibrils in the subepithelial ECM of injured corneas. Moreover, 2-*O*-sulfated heparan compounds significantly inhibited corneal infection in immunocompromised hosts, by a clinical keratitis isolate of *S. pneumoniae*, and also when topically administered in a therapeutic manner. These findings suggest that the administration of nonanticoagulant 2-*O*-sulfated heparan compounds may represent a plausible approach to the treatment of *S. pneumoniae* keratitis.

## INTRODUCTION

Attachment of pathogens to host tissues is thought to be an important mechanism that promotes pathogenesis.^1^ Many microbial pathogens interact with host extracellular matrix (ECM) components to promote their adhesion and invasion. The importance of pathogen-ECM interactions in pathogenesis is supported by studies showing that the deletion of genes for bacterial ECM binding proteins reduces virulence in animal models of infection.^2–4^ Studies showing that immunization against the bacterial ECM binding proteins generates protective immunity also suggest that ECM binding is a virulence activity.^5–7^ However, because many bacterial ECM binding proteins bind to multiple ECM components and some even have endogenous functions, these studies do not clearly establish that binding to a particular ECM molecule is essential for infection. An approach where the ECM binding activity is specifically inhibited in vivo is required to clearly establish the importance of a particular bacterial pathogen-ECM interaction in pathogenesis.

Fibronectin (FN) is frequently targeted by bacterial pathogens for their adhesion to host tissues. FN is a large multidomain glycoprotein that contains binding sites for integrins, ECM components, and microbial adhesins, among others.^8^ FN is a ubiquitous component of interstitial matrices but not an intrinsic component of basement membranes. However, FN fibrils are found immediately adjacent to the epithelial basement membrane in several tissues,^9–11^ including the cornea.^12–14^ A recent study demonstrated that basement membrane components induce FN fibrillogenesis,^15^ partly explaining how FN fibrils are assembled adjacent to basement membranes, but why this process does not happen uniformly in all basement membranes is not known.

So far, over 100 Gram-positive and Gram-negative FN-binding proteins have been identified,^16^ suggesting that FN binding activity has arisen through convergent evolution because it is advantageous for the survival of bacteria in the host. The majority of bacterial FN-binding proteins bind to the N-terminal type I modules 1–5 of FN, which contain the HepI heparin (HP) binding domain. However, several bacterial FN-binding proteins bind to the C-terminal type III repeats in FN in an HP-inhibitable manner, suggesting that they bind to the HepII HP binding domain in the C-terminus. Examples include *Streptococcus pneumoniae* PavA and PavB,^17^ *Staphylococcus epidemidis* Embp,^18,19^ *Salmonella typhimurium* ShdA,^20^ and *Bartonella henselae* Pap31.^21^ The reason why bacterial pathogens attach to different HP binding domains in FN is not understood, but by doing so, they may avoid competing for attachment sites during polymicrobial infections. Furthermore, because both HepI and HepII domain interactions facilitate FN fibrillogenesis,^22–24^ which is an important process in tissue repair, binding of bacterial FN-binding proteins to HepI and HepII domains might interfere with FN fibrillogenesis, delay wound repair, and promote pathogenesis. Indeed, a peptide corresponding to the FN binding domain in *Streptococcus pyogenes* F1 protein, which binds to the N-terminal HepI domain, inhibits FN assembly by fibroblasts.^25^

Similar to FN, many bacterial pathogens bind to HS chains of HS proteoglycans (HSPGs). Studies in the 1970s showed that HP potently inhibits the attachment of *Neisseria meningitidis*^26^ and *Chlamydia trachomatis*^27^ to host cells. Because HS and HP share a common biosynthetic pathway and HP is structurally related to the protein-binding, sulfated regions of HS, these findings suggest that the HS moiety of cell surface HSPGs is exploited for bacterial adhesion. Indeed, we now know that HS interactions are used not only for bacterial attachment but also for bacterial invasion of host cells and systemic dissemination. Bacterial pathogens can use cell surface HS as a direct receptor or coreceptor for their attachment, entry, and dissemination.^28,29^ HS is a polydisperse linear polysaccharide that is ubiquitously expressed on the cell surface and in the ECM. HS is synthesized on HSPG core proteins in a nontemplate-driven process.^30^ The HS backbone is polymerized by the addition of β1,4-linked glucuronic acid (GlcA) and α1,4-linked *N*-acetylglucosamine (GlcNAc) units in alternating sequence to the nonreducing end of the growing polysaccharide. Concomitant with chain elongation, HS is modified by various sulfotransferases and an epimerase to generate a complex polysaccharide containing GlcNAc and *N*-sulfated glucosamine (GlcNS) residues, GlcA and iduronic (IdoA) acids, as well as *O*-sulfate groups at the C2 position of uronic acids, C6 of glucosamine, and in rare cases, C3 position of glucosamine. The interaction of HS with proteins depends largely on the sulfate modifications, rotations around the glycosidic linkages, conformational flexibility of pyranose rings in IdoA, and chain length.

We recently examined if *S. pneumoniae* binds to syndecan-1 and uses this interaction for its corneal infection because syndecan-1 is the major HSPG of epithelial cells, a cell type frequently targeted for pathogenesis. *S. pneumoniae* has also been suggested to interact with cell surface HSPGs either directly^31^ or indirectly.^32,33^ Consistent with the idea that syndecan-1 is a receptor for bacterial attachment, we found that deletion of syndecan-1 significantly reduces *S. pneumoniae* virulence in mouse corneas.^12^ However, *S. pneumoniae* did not bind to syndecan-1 and gene silencing of syndecan-1 had no effect on bacterial adhesion to corneal epithelial cells. Instead, the hyposusceptible phenotype of syndecan-1 knockout mice in *S. pneumoniae* corneal infection was traced to a marked reduction of FN fibrils in the corneal basement membrane that serve as *S. pneumoniae* attachment sites. Under normal conditions, epithelial syndecan-1 drives FN assembly in corneal basement membranes and the absence of syndecan-1 in mutant mice led to decreased adhesive FN fibrils. We also serendipitously found that HS chains of syndecan-1 strongly inhibit *S. pneumoniae* corneal infection.^12^ In this study, we investigated how HS inhibits *S. pneumoniae* corneal infection. Our results demonstrate that *S. pneumoniae* binds directly to FN fibrils assembled by corneal epithelial cells and that heparan compounds containing 2-*O*-sulfate groups protect against *S. pneumoniae* corneal infection by specifically interfering with bacterial adhesion onto FN fibrils.

## Materials and methods

### Materials

Todd–Hewitt broth, yeast extract, Epilife culture medium, FBS, human corneal growth supplement, and Verso 1-step quantitative reverse transcription polymerase chain reaction (RT-qPCR) kit were purchased from Thermo Fisher. Trypticase soy agar (TSA) plates with 5% sheep blood were from BD Biosciences. Porcine mucosal HS, heparin (HP), *N*-desulfated heparin (NDS-HP), 2-*O*-desulfated heparin (2ODS-HP), and 6-*O*-desulfated heparin (6ODS-HP) were from Neoparin. Disaccharide composition analyses confirmed that 100% of *N*-sulfates, 98% of 2-*O*-sulfates, and 76% of 6-*O*-sulfates were removed from NDS-HP, 2ODS-HP, and 6ODS-HP, respectively (Supporting Information: Table S1). Bovine tracheal CS-A and the Duolink Proximity Ligation Assay (PLA) kit were purchased from Sigma. Oligonucleotide primers were from IDT. RNeasy Plus Mini Kit was from Qiagen. Heparinase I-treated HS and heparinase III-treated HS were generated by heparinase I and heparinase III (R&D System) digestion of HS, respectively. Briefly, 20 μg of HS were digested with 5 μg/mL heparinase I or heparinase III at 37°C, added sequentially at 3 h intervals. Digestion was complete after 6 h and was stopped by heating at 100°C for 30 min to inactivate the heparinases. Heparosan (H) from *Escherichia coli* K5, *N*-sulfated H (NS-H) generated chemoenzymatically with purified recombinant *N*-sulfotransferase and sulfate donor 3′-phosphoadenosine 5′-phosphosulfate (PAPS), and *N*- and 2-*O*-sulfated H (NS2OS-H) generated with *N*-sulfotransferase, HS 2-*O*-sulfotransferase, and PAPS were prepared and characterized in Dr. Jian Liu’s lab as previously described.^34,35^ Size-defined HP oligosaccharides, with the main disaccharide composition of trisulfated IdoA2S-GlcNS6S, were gifts from Dr. John Gallagher (University of Manchester, Manchester, UK). All other materials were purchased from Thermo Fisher, Sigma, or VWR.

### Immunochemicals

Rat anti-mouse Ly6G antibody (1A8, 100 μg/mouse i.p. 24 and 0.5 h before infection for neutrophil depletion) was purchased from BioXCell. Rabbit anti-FN antibody (5 μg/mL for immunostaining and PLA) was purchased from Abcam or Sigma. Rabbit anti-LM 5 antibody (10 μg/mL for immunostaining and PLA) was from Bioss. Mouse anti-α-smooth muscle cell actin (αSMA) antibody (1A4, 15 μg/mL for IHC) was from Sigma. Alexa 647-conjugated rat anti-nidogen antibody (ELM1, 2 μg/mL for immunostaining) was from Santa Cruz. Rabbit antiperlecan antibody (1:100 for immunostaining) was a gift from Dr. Merton Bernfield (Boston Children’s Hospital). Human anti-poly-*N*-acetylglucosamine (PNAG) antibody (17 μg/mL for immunostaining and PLA) was a gift from Dr. Gerald Pier (Harvard Medical School). Fluorophore-conjugated secondary antibodies were purchased from Invitrogen/Thermo Fisher.

### Bacteria

*S. pneumoniae* strains TIGR4 (serotype 4) and L82016 (serotype 6B),^36^ and clinical keratitis isolate IDI30/44 (sequence type 558, serotype 35B)^37^ were grown on 5% sheep blood TSA plates or in 4 mL of Todd–Hewitt broth supplemented with 0.5% yeast extract (THY broth) in glass tubes without agitation to mid-log growth phase. The bacterial concentration was approximated by measuring absorbance at OD600 nm. After washing, the concentration was adjusted to the desired concentration for the in vitro adhesion assays and in vivo corneal infection assays as indicated. Viable *S. pneumoniae* was enumerated by colony formation on 5% sheep blood TSA plates to determine the exact infectious inoculum.

### S. pneumoniae corneal infection

Both female and male BALB/cJ mice were used at an age of 5–10 weeks. Mice were maintained in microisolator cages under specific pathogen-free conditions in a 12-h light/dark cycle and fed a basal rodent chow ad libitum. Use of *S. pneumoniae* and *S. pneumoniae* corneal infection experiments were approved by the Institutional Biosafety Committee (IBC) and Institutional Animal Care and Use Committee (IACUC) of Boston Children’s Hospital and complied with federal guidelines for research with experimental animals. Four vertical scratches were made with a 26G needle in one of the corneas of each anesthetized mouse without penetrating beyond the superficial stroma.

Injured corneas were infected topically with various doses of *S. pneumoniae* without or with test reagents. At the indicated times postinfection (pi), mice were euthanized, and eyes were enucleated and transected posterior to the corneal limbus under a dissecting microscope. The bacterial burden in isolated corneas was determined by homogenizing in THY broth containing 0.1% (v/v) Triton X-100, plating out serial dilutions onto blood agar plates, and counting the number of colonies. In some experiments, mice were made neutropenic by injecting 100 μg of anti-Ly6G antibodies at 24 and 0.5 before injury and infection to test the protective effects of heparan compounds on *S. pneumoniae* corneal infection in immunocompromised hosts.

### RNA extraction from cornea and RT-qPCR

Total RNA was isolated by RNAeasy Plus mini kit (Qiagen), and the concentration was determined by a NanoDrop spectrophotometer (Thermo Scientific). RT-qPCR was performed with 20 ng of template RNA using 1 μM of primers for tumor necrosis factor (TNF)-α (F: GGTGCCTATGTCTCAGCCTCTT, R: GCCATAGAACTGATGAGAGGGAG), transforming growth factor β1 (TGFβ1) (F: TGATACGCCTGAGTGGCTGTCT, R: CACAAGAGCAGTGAGCGCTGAA), VEGF (F: CTGCTGTAACGATGAAGCCCTG, R: GCTGTAGGAAGCTCATCTCTCC), and glyceraldehyde-3-phosphate dehydrogenase (GAPDH) (F: CATCACTGCCACCCAGAAGACTG, R: ATGCCAGTGAGCTTCCCGTTCAG), and a Verso 1-step RT-qPCR Kit (Thermo Fisher) on a CFX96TM Real-Time System (Bio-Rad). Target gene expression was normalized to the housekeeping gene GAPDH using quantification cycle (Δ*C*_q_) between the target genes and GAPDH.

### Immunostaining of corneal sections

Enucleated eyes were fixed in 4% paraformaldehyde/PBS for 4 h at room temperature, embedded in paraffin, and sectioned. Eye sections (5 μm) were deparaffinized in xylene, 100%−50% ethanol gradient, hydrated in PBS, microwaved in defrost mode for 6 min (×2) in 50 mM Tris, pH 8.8 and 1 mM EDTA for antigen retrieval, and background signals were quenched in 100 mM NH_4_Cl. Sections were then blocked with 10% nonimmune serum of secondary antibodies in PBS and immunostained with the indicated specific antibodies and corresponding secondary antibodies. Where fluorophore-conjugated primary antibodies were used, sections were blocked with 3% BSA in PBS. Images were captured with the Zeiss Axiovert 40 CFL microscope, and pictures were taken with the AxioCam MRm high-resolution camera. Adobe Photoshop was used to process the acquired images.

### Decellularization and immunostaining of decellularized ECM

A6(1) mouse corneal epithelial cells were seeded at 30% confluency in eight-well chamber slides for staining and PLA, and 48-well plates for adhesion assays. At 2 days postconfluency, cells were washed twice with PBS and three times with 100 mM Na_2_HPO_4_, pH 9.6, containing 2 mM MgCl_2_ and 2 mM EGTA. Cells were then lysed by incubating twice in 8 mM Na_2_HPO_4_, pH 9.6, containing 1% NP-40 for 15 min each at 37°C. The decellularized A6(1) ECM was washed three times with 10 mM Na_2_HPO_4_, pH 7.5, containing 300 mM KCl, and then washed five times with deionized H_2_O. For immunostaining, the decellularized ECM was fixed with 4% formaldehyde, blocked with 3% BSA in PBS, and immunostained with primary antibodies and Alexa 488- or Alexa 594-conjugated secondary antibodies or with Alexa 647-conjugated primary antibodies. Images were captured with a Zeiss Axiovert 40 CFL microscope and AxioCam high-resolution camera. Adobe Photoshop was used to process the acquired images.

### PLA

PLA was used to determine the binding interaction between *S. pneumoniae* and components of the ECM assembled by A6(1) corneal epithelial cells. The decellularized ECM of confluent A6(1) cells was incubated with test reagents for 30 min, and then incubated with *S. pneumoniae* TIGR4 for 1 h at room temperature. The TIGR4-bound decellularized ECM was washed three times with PBS and processed for PLA per the manufacturer’s instructions. Briefly, the TIGR4-bound decellularized ECM was blocked with the manufacturer’s blocking solution, incubated overnight with anti-FN or anti-laminin and anti-PNAG antibodies at 4°C, washed twice for 5 min at room temperature, and incubated with plus and minus PLA probes (2° antibodies with 5′ and 3′ oligonucleotides) for 1 h at 37°C. Washed samples were incubated in ligation buffer containing DNA ligase for 30 min at 37°C to form closed, circular DNA templates, washed twice for 5 min at room temperature, and incubated in amplification solution containing DNA polymerase for 100 min at 37°C to generate concatemeric sequences during rolling circle amplification. After washing, red fluorescence PLA signals were captured on Zeiss Axiovert 40 CFL microscope and AxioCam MRm high-resolution camera.

### *S. pneumoniae* adhesion

Decellularized ECM of A6(1) cells were pretreated without or with test reagents for 30 min and incubated with *S. pneumoniae* TIGR4 (4–8× 10^5^ cfu) in 200 μL PBS for 1 h at room temperature. Unbound TIGR4 were removed by three washes with PBS, and adherent TIGR4 bacteria were recovered by incubating with 0.1% Triton X-100/THY broth for 20 min at room temperature and vigorous pipetting. Serial dilutions were plated onto blood agar plates to count TIGR4 colonies.

### Data analysis

Data are expressed as scatterplots, bar graphs, or line graphs with mean ± SEM. Statistical significance between experimental and control groups was analyzed by two-tailed unpaired Student’s *t* test and between multiple groups by analysis of variance followed by Dunnett’s post-hoc test using GraphPad Prism software (version 9.5.0). *p* < 0.05 were determined to be significant.

## RESULTS

### Sulfated domains in HS and HP inhibit *S. pneumoniae* corneal infection

*S. pneumoniae* is a leading cause of sight-threatening corneal infections. At several clinical institutions, *S. pneumoniae* has been found to account for over 33% of bacterial keratitis cases.^38–42^ Intact, healthy corneas are remarkably resistant to *S. pneumoniae* infection even at a very large inoculum (≥10^9^ cfu). Enucleated eyes also resist infection when submerged ex vivo into a suspension of *Pseudomonas aeruginosa*.^43^ These observations indicate that unbound components of the tear fluid are not essential for strong resistance and that the stratified corneal epithelium provides a highly efficient physical barrier to infection. Indeed, corneal epithelial abrasion caused by trauma, surgery, or contact lens wear significantly increases the susceptibility to infection,^39^ suggesting that bacterial interactions with the subepithelial ECM are critical for pathogenesis.

**FIGURE 1:**
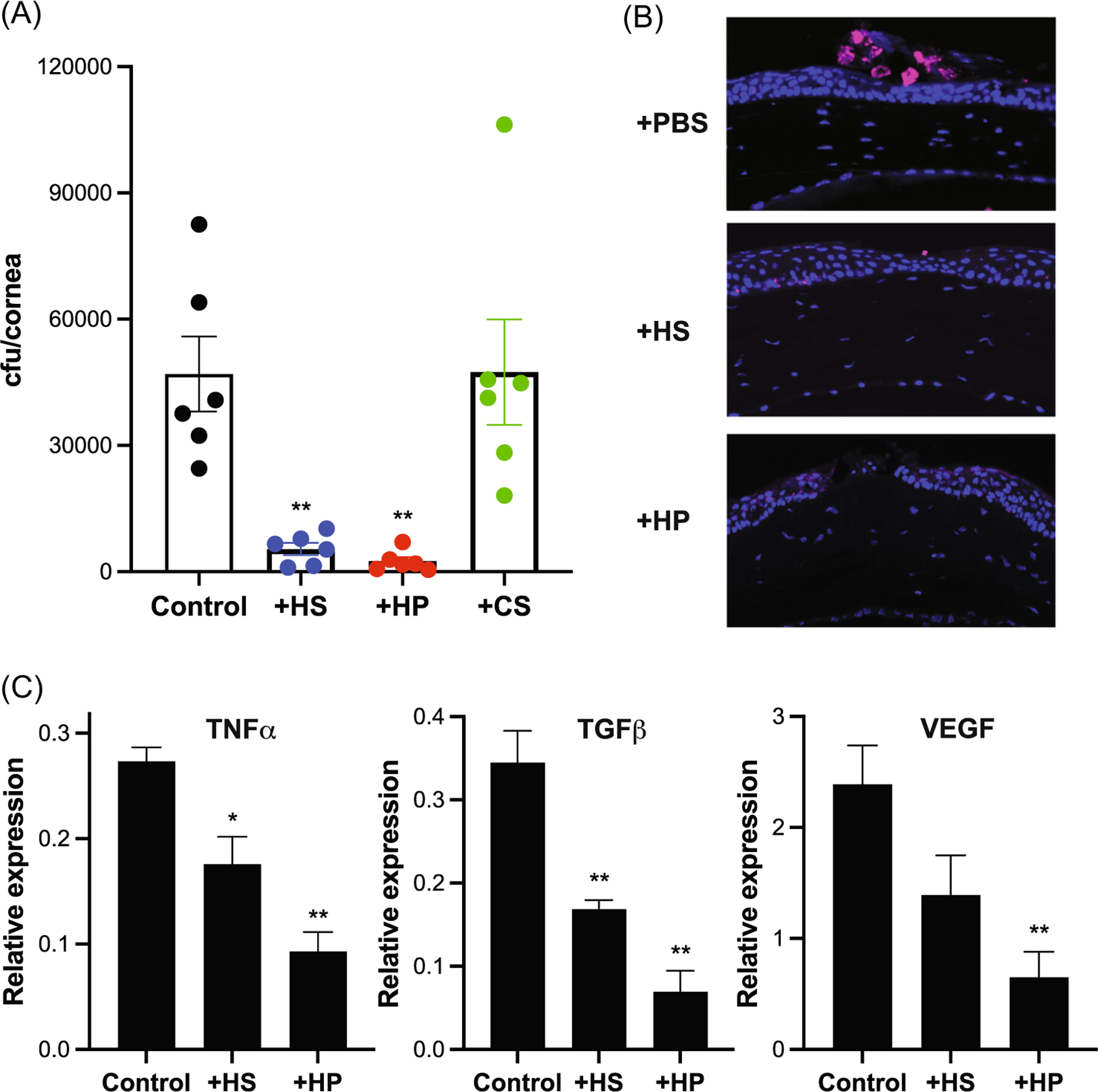
HS and HP protect against *Streptococcus pneumonia* corneal infection. Injured Wt mouse corneas were topically coinfected with 10^8^ cfu of *S. pneumoniae* strain TIGR4 and PBS (control) or 200 ng of HS, HP, or CS and assessed for parameters of keratitis at 6 h pi. (A) Corneal bacterial burden (mean ± SE, *n* = 6, ^**^p < 0.01, ANOVA with Dunnett’s multiple comparison test). (B) Corneal sections immunostained with anti-PNAG antibodies (original magnification, ×200). (C) mRNA levels of TNF-α, TGFβ1, and VEGF by RT-qPCR. Δ*C*_q_ values normalized to GAPDH are shown as “Relative Expression” (mean ± SE, *n* = 3, *p < 0.05, ^**^p < 0.01). ANOVA, analysis of variance; CS, chondroitin sulfate; GAPDH, glyceraldehyde-3-phosphate dehydrogenase; HP, heparin; HS, heparan sulfate; mRNA, messenger RNA; pi, postinfection; PNAG, poly-*N*-acetylglucosamine; RT-qPCR, quantitative reverse transcription polymerase chain reaction; TGFβ1, transforming growth factor β1; TNF, tumor necrosis factor; Wt, wild type.

We first compared the effects of HS and HP on *S. pneumoniae* infection of injured mouse corneas. The corneal epithelium was injured by making four vertical scratches with a 26G needle. Abraded corneas were topically infected with *S. pneumoniae* strain TIGR4 (serotype 4) with parallel administration of 200 ng HS, HP, or chondroitin sulfate (CS) and assessed for parameters of bacterial keratitis. Compared to injured corneas infected with TIGR4 and vehicle (PBS), the corneal bacterial burden was reduced by 9- and 19-fold in corneas infected with TIGR4 and HS and HP, respectively, at 6 h pi (Figure 1A). Topical administration of CS had no inhibitory effect on TIGR4 infection (Figure 1A). To visualize infection, we immunostained infected corneal sections with a human monoclonal antibody against PNAG, a conserved microbial cell surface polysaccharide expressed by many bacterial pathogens, including *S. pneumoniae*.^44,45^ Immunostaining with anti-PNAG showed that PNAG+TIGR4 primarily infects at the site of injury, which was markedly reduced when corneas were given HS or HP (Figure 1B). Inflammatory and tissue repair responses stimulated by *S. pneumoniae* infection, but not by injury alone, were also significantly inhibited by HS and HP, as mRNA levels of TNF-α, TGFβ, and VEGF (Figure 1C) and αSMA-positive corneal epithelial cells were significantly reduced (Supporting Information: Figure S1).

**FIGURE 2:**
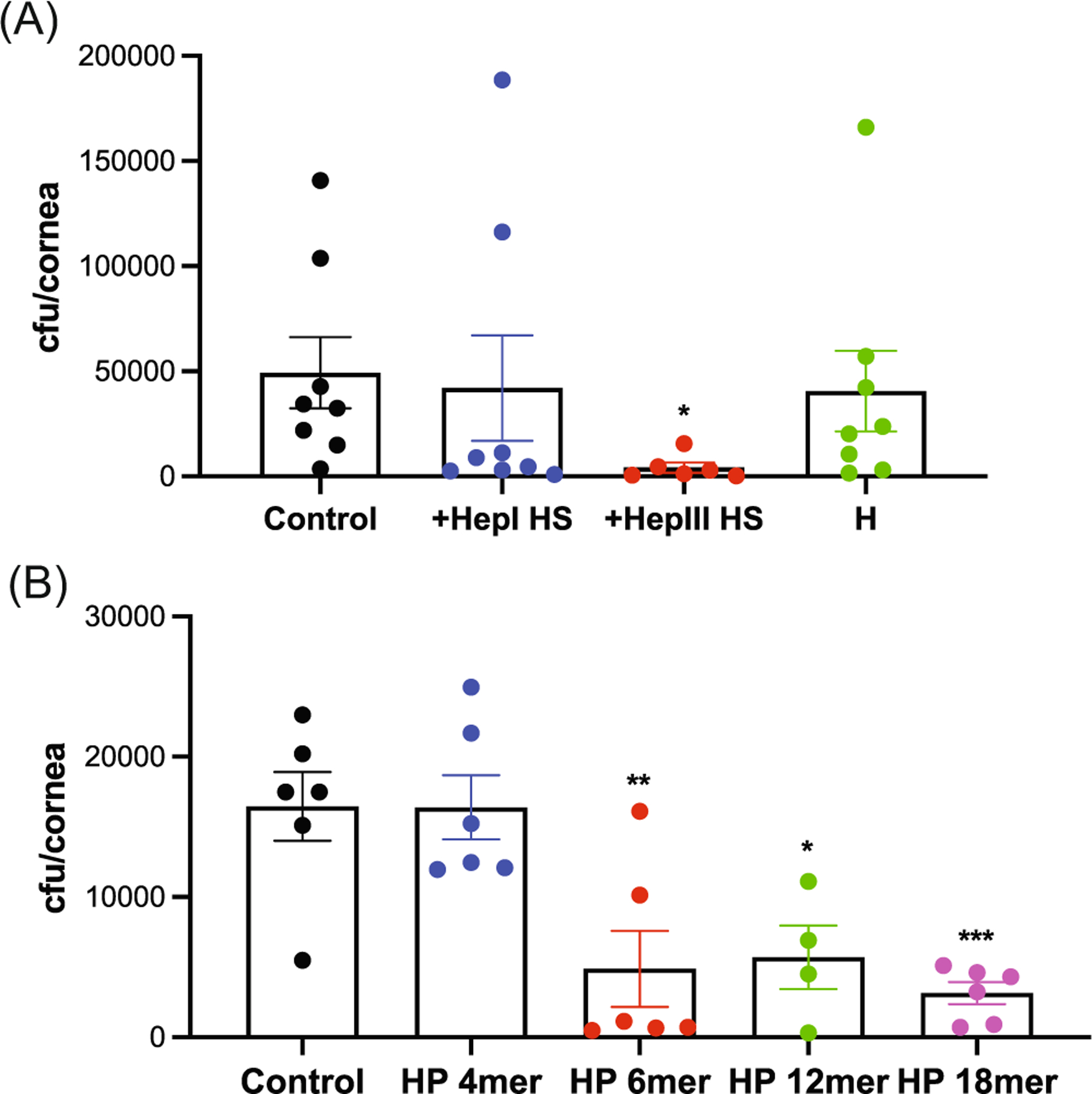
Sulfated heparan compounds inhibit *Streptococcus pneumoniae* corneal infection. (A) Injured corneas were topically coinfected with 10^8^ cfu of *S. pneumoniae* TIGR4 and PBS (control) or 200 ng of heparinase I-digested HS (HepI HS), heparinase III-digested HS (HepIII HS), or heparosan (H) and the corneal bacterial burden was measured at 6 h pi (mean ± SE, *n* = 6–8, *p < 0.05). (B) Injured corneas were topically coinfected with 10^8^ cfu of TIGR4 and HP 4-mer (800 ng), 6-mer (500 ng), 12-mer (400 ng), or 18-mer (200 ng) oligosaccharides and the corneal bacterial burden was assessed at 6 h pi (mean ± SE, *n* = 4–6, *p < 0.05, ^**^p < 0.01, ^***^p < 0.001). HP, heparin; HS, heparan sulfate; pi, postinfection.

HS significantly inhibits *S. pneumoniae* corneal infection when coadministered with bacteria but not when preincubated with bacteria and washed away before infection.^12^ Furthermore, HS chains of syndecan-1 do not bind to *S. pneumoniae*,^12^ suggesting that HS affects the host, and not bacteria, when attenuating *S. pneumoniae* corneal infection. However, little is known about the structural features of HS that inhibit *S. pneumoniae* corneal infection. To pursue the essential structural features, we first tested the effects of heparosan (H) and HS fragments generated by heparinase I and III digestion. Heparinase I and III digest sulfated and low-sulfated domains of HS, releasing low-sulfated and sulfated HS fragments, respectively.^46^ H is a polysaccharide component of *E. coli* K5 capsule that has a repeating disaccharide unit of -GlcA-GlcNAc- and is identical in structure to unmodified HS.^47,48^ Sulfated fragments of HS released by heparinase III digestion significantly reduced the corneal bacterial burden by 12-fold when coadministered with *S. pneumoniae* TIGR4, whereas heparinase I-digested HS and H did not (Figure 2A), indicating that sulfated motifs in HS are critical for inhibition of *S. pneumoniae* corneal infection. We also tested the effects of HP oligosaccharides to determine the minimum size of HP necessary to inhibit *S. pneumoniae* corneal infection. HP 6-, 12-, and 18-mer significantly decreased the corneal bacterial burden by 65%–81% (Figure 2B). HP oligosaccharides shorter than 6-mer did not inhibit infection, indicating that a length of at least 6-mer is required for HP oligosaccharides to significantly inhibit *S. pneumoniae* corneal infection.

### HS and HP inhibit the direct binding of *S. pneumoniae* to FN fibrils assembled by corneal epithelial cells

**FIGURE 3:**
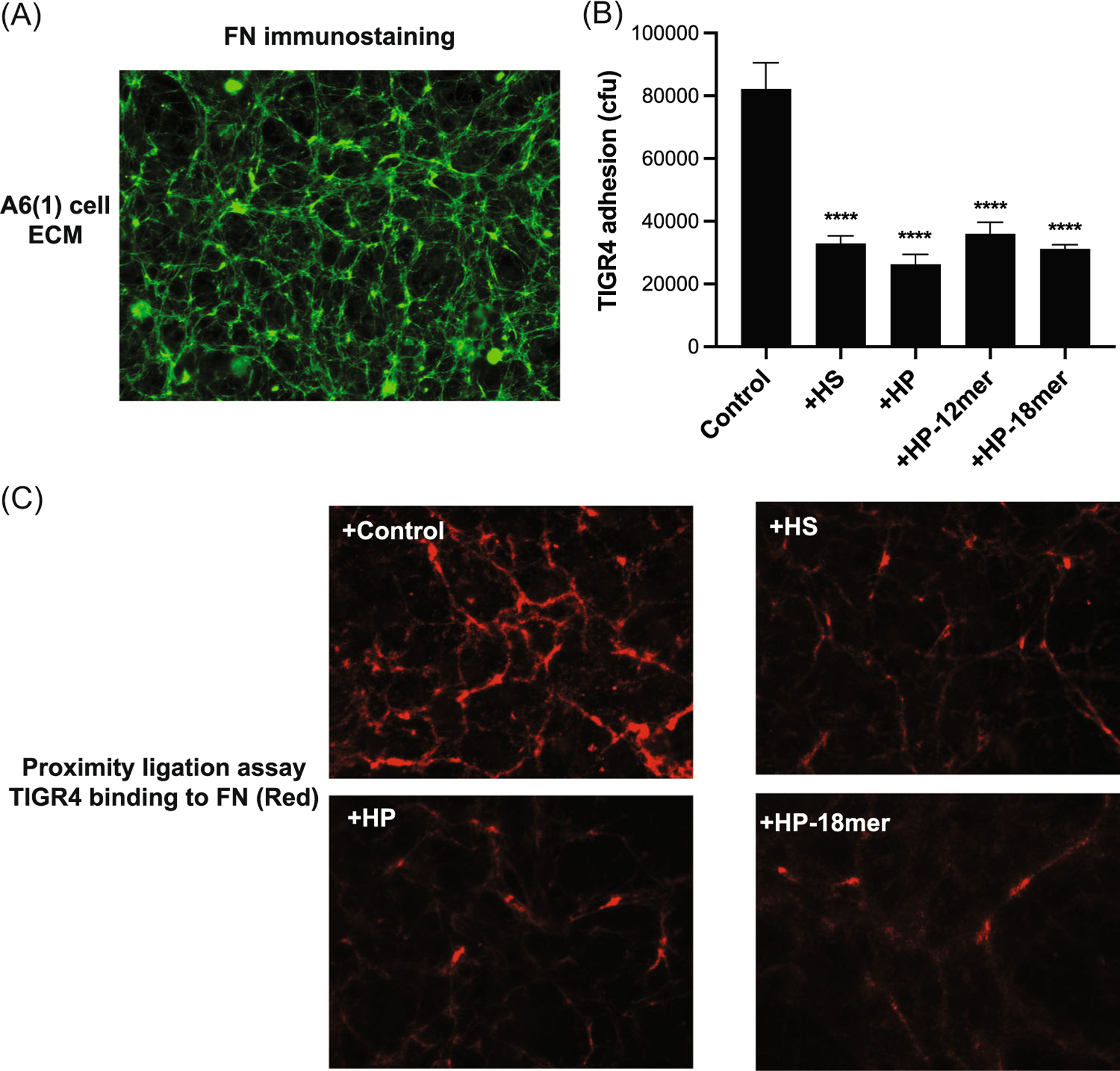
HS, HP, and HP oligosaccharides inhibit *Streptococcus pneumoniae* adhesion onto FN fibrils in the ECM of cultured corneal epithelial cells. A6(1) corneal epithelial cells were seeded at 30% confluence and cultured for 6 days and decellularized. (A) The decellularized ECM was fixed with 4% formaldehyde, blocked, and immunostained with anti-FN and Alexa 488 secondary antibodies. Images were captured with a Zeiss Axiovert 40 CFL microscope and AxioCam high-resolution camera (original magnification, ×200). (B) The decellularized ECM of confluent A6(1) corneal epithelial cells was incubated with PBS (control) or 5 μg/mL of HS, HP, HP 12-mer, or HP 18-mer for 30 min and 5× 10^5^ cfu of *S. pneumoniae* TIGR4 was added and incubated for another 1 h. (B) Attached bacteria were quantified by plating out serial dilutions of detergent extracts and counting the number of colonies (mean ± SE, *n* = 5, ^****^p < 0.0001, ANOVA). (C) Binding of *S. pneumoniae* to FN fibrils in the decellularized ECM of A6(1) cells treated without or with 5 μg/mL HS, HP, or HP 18-mer was assessed by PLA using anti-FN and anti-PNAG antibodies. Red indicates where the distance between TIGR4 and FN is ≤40 nm (original magnification, ×400). ANOVA, analysis of variance; ECM, extracellular matrix; FN, fibronectin; HP, heparin; HS, heparan sulfate; PNAG, poly-*N*-acetylglucosamine.

Previous in vivo studies have suggested that *S. pneumoniae* binds to FN fibrils in the basement membrane when infecting injured corneas.^12^ Furthermore, the FN binding site in HS is resistant to heparinase III digestion, whereas it is susceptible to heparinase I digestion.^49^ Together with our data showing that HS inhibition of *S. pneumoniae* corneal infection is sensitive to heparinase I, but not to heparinase III digestion, these findings suggest that HS and HP are inhibiting infection by interfering with *S. pneumoniae* interactions with FN. To further pursue how HS and HP protect against *S. pneumoniae* corneal infection, we examined if corneal epithelial cells in culture assemble an ECM resembling the corneal basement membrane and if *S. pneumoniae* interacts with FN fibrils in the decellularized ECM of corneal epithelial cells. A6(1) mouse corneal epithelial cells^50,51^ were cultured to confluency and decellularized by detergent and high pH.^52^ Decellularization does not affect the organization of the underlying ECM.^53^ Immunostaining showed that FN fibrils and other major basement membrane components, such as laminin and nidogen, are present in the ECM assembled by corneal epithelial cells (Figure 3A and Supporting Information: Figure S2).

We tested whether heparan compounds that attenuate *S. pneumoniae* corneal infection also inhibit *S. pneumoniae* adhesion onto the decellularized ECM of corneal epithelial cells. In titration assays, pretreatment of the decellularized ECM of A6(1) corneal epithelial cells with HS inhibited *S. pneumoniae* TIGR4 adhesion in a dose-dependent manner, reaching maximal inhibition at 5 μg/mL (Supporting Information: Figure S3). Based on these data, the decellularized ECM of A6(1) cells were treated with 5 μg/mL HS, HP, or HP oligosaccharides for 30 min, incubated with TIGR4 for 1 h, and bacterial adhesion onto the ECM was quantified. HS and HP significantly inhibited TIGR4 adhesion by 60% and 68%, whereas HP 12-mer and HP 18-mer significantly decreased TIGR4 adhesion by 56% and 62% compared to the control (Figure 3B). These results suggest that heparan compounds protect against *S. pneumoniae* corneal infection by inhibiting bacterial adhesion to the subepithelial ECM exposed in injured corneas.

**FIGURE 4:**
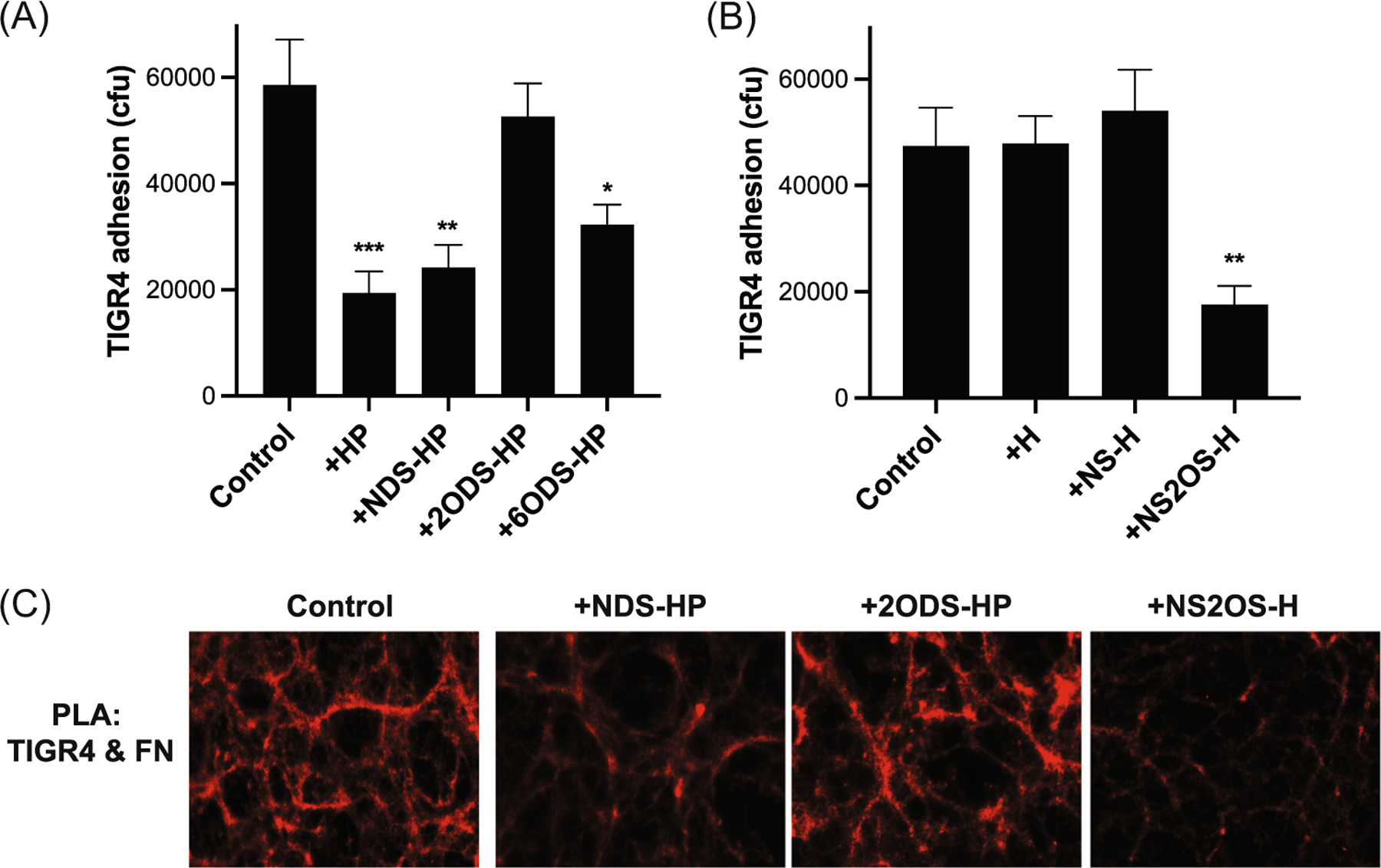
2-*O*-sulfated heparan compounds inhibit *Streptococcus pneumoniae* adhesion onto FN fibrils in the ECM of A6(1) corneal epithelial cells. The decellularized ECM of A6(1) corneal epithelial cells was incubated with PBS (control) or (A) 5 μg/mL of HP, NDS-HP, 2ODS-HP, or 6ODS-HP or (B) heparosan (H), NS-H, or NS2OS-H for 30 min and 8 × 10^5^ cfu of *S. pneumoniae* TIGR4 were added and incubated for another 1 h. Attached bacteria were quantified by plating out serial dilutions of detergent extracts and counting the number of colonies (mean ± SE, n = 4, *p < 0.05, ^**^p < 0.01, ^***^p < 0.001). (C) Binding of *S. pneumoniae* to FN fibrils in the ECM of A6(1) cells in the absence or presence of 5 μg/mL NDS-HP, 2ODS-HP, or NS2OS-H was assessed by PLA (original magnification, ×400). 2ODS-H, 2-*O*-desulfated heparin; 6ODS-HP, 6-*O*-desulfated heparin; ECM, extracellular matrix; FN, fibronectin; HP, heparin; HS, heparan sulfate; NDS-HP, *N*-desulfated heparin; NS-H, *N*-sulfated H; NS2OS-H, *N*- and 2-*O*-sulfated H; PLA, proximity ligation assay; PNAG, poly-*N*-acetylglucosamine.

We next performed PLAs to establish that *S. pneumoniae* binds directly to FN fibrils in the corneal epithelial ECM and that heparan compounds inhibit *S. pneumoniae* adhesion onto the corneal epithelial ECM by interfering with FN binding. PLA uses specific antibodies against molecules of interest, oligonucleotide-labeled secondary antibodies, and DNA polymerase-mediated amplification of fluorescent signal to detect physical interactions between molecules.^54^ Decellularized matrices of A6(1) cells pretreated with HS, HP, or HP 18-mer were incubated with *S. pneumoniae* TIGR4 for 1 h. The interaction between TIGR4 and FN was determined using anti-PNAG and anti-FN antibodies and PLA that produces a red fluorescent signal when the physical distance between *S. pneumoniae* and FN is ≤40 nm. PLA showed a fibrillar pattern of TIGR4 bound to FN in the control group (Figure 3C), resembling fibrillar FN signals seen in the decellularized ECM immunostained for FN (Figure 3A). These data strongly suggest that *S. pneumoniae* binds directly to FN fibrils assembled by corneal epithelial cells. Addition of HS, HP, or HP 18-mer markedly reduced the red PLA signal, indicating that heparan compounds inhibit the direct interaction between TIGR4 and FN (Figure 3C). Furthermore, very little PLA signal was generated when probed for TIGR4 binding to laminin (Supporting Information: Figure S4), despite the fact that abundant laminin polymers are deposited in the ECM of A6(1) cells (Supporting Information: Figure S2). Together, these results suggest that TIGR4 binds directly and selectively to FN fibrils in the corneal epithelial ECM. However, dense, node-like PLA signals remained even in the presence of inhibitory heparan compounds (Figure 4C). Similar dense, node-like structures were also seen when the decellularized ECM was immunostained for FN (Figure 3A). These areas of dense PLA and immunostained FN signals might represent sites where the concentration of FN is extremely high, such as at nucleation sites of FN fibrillogenesis.^55^

### 2-*O*-sulfated HS motifs inhibit *S. pneumoniae* adhesion onto FN fibrils assembled by corneal epithelial cells

We next investigated the impact of HS and HP sulfation on the inhibition of *S. pneumoniae* adhesion because sulfate modifications govern HS and HP functions.^56^ We tested the effects of chemically desulfated HP and chemoenzymatically sulfated H compounds. *S. pneumoniae* TIGR4 adhesion onto decellularized matrices of A6(1) cells pretreated with NDS-HP and 6ODS-HP was significantly reduced by 59% and 45%, respectively, compared to the control group pretreated with PBS (Figure 4A). However, the addition of 2ODS-HP did not inhibit TIGR4 adhesion (Figure 4A). Consistent with these observations, pretreatment of the decellularized ECM with NS2OS-H significantly inhibited TIGR4 adhesion by 62% compared to the control, but pretreatment with nonsulfated H and NS-H did not (Figure 4B). Corroborating these findings, PLA showed that NDS-HP and NS2OS-HP markedly reduce red PLA signals corresponding to TIGR4 bound to FN, whereas 2ODS-HP had no noteworthy inhibitory effect (Figure 4C). Altogether, these results indicate that *S. pneumoniae* directly attaches to FN fibrils in the ECM of corneal epithelial cells, and that HS and HP inhibit this interaction via 2-*O*-sulfated motifs.

### 2-*O*-sulfated HS domains inhibit *S. pneumoniae* corneal infection

**FIGURE 5:**
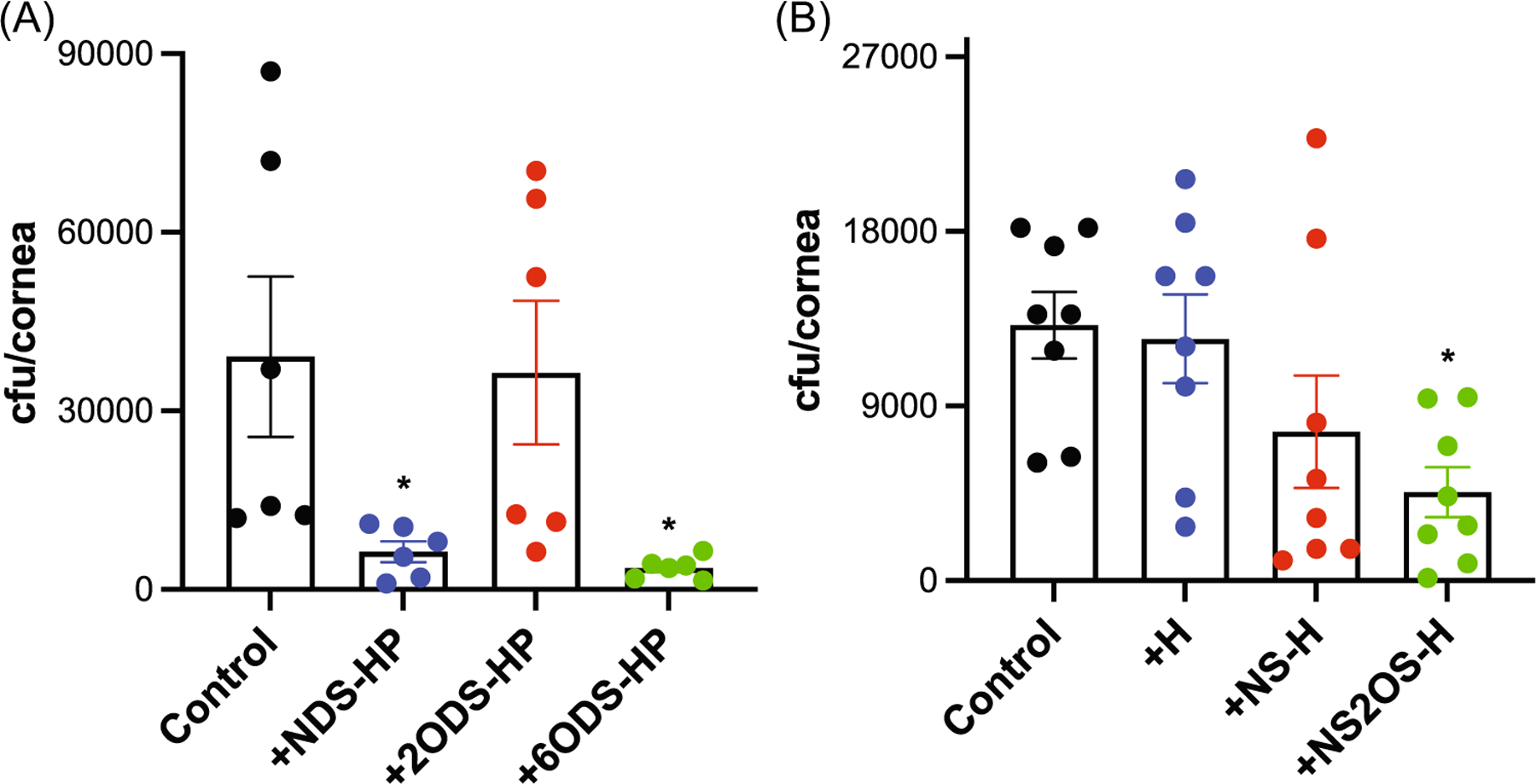
2-*O*-sulfated heparan compounds attenuate *Streptococcus pneumoniae* corneal infection. Injured mouse corneas were coinfected with 10^8^ cfu of *S. pneumoniae* TIGR4 and PBS (control) or (A) 300 ng NDS-HP, 2ODS-HP, or 6ODS-HP (mean ± SE, *n* = 6, *p < 0.05) or (B) 300 ng of H, NS-H, or NS2OS-H and the corneal bacterial load was measured at 6 h pi (mean ± SE, *n* = 8, *p < 0.05). 2ODS-HP, 2-*O*-desulfated heparin; 6ODS-HP, 6-*O*-desulfated heparin; HP, heparin; NDS-HP, *N*-desulfated heparin; NS-H, *N*-sulfated H; NS2OS-H, *N*- and 2-*O*-sulfated H.

We next tested whether 2-*O*-sulfated HS motifs are also critical for the inhibition of *S. pneumoniae* corneal infection in vivo. Injured mouse corneas were topically infected with *S. pneumoniae* TIGR4 and chemically desulfated HP compounds or chemoenzymatically sulfated H compounds, and their effects on corneal bacterial counts were assessed. Removal of *N*- and 6-*O*-sulfates had no significant effect on the ability of HP to inhibit TIGR4 corneal infection (Figure 5A). Topical administration of NDS-HP decreased the corneal bacterial burden by 84%, whereas 6ODS-HP reduced it by 91%. However, 2-*O*-desulfation abrogated the ability of HP to inhibit bacterial adhesion (Figure 5A). Consistent with these data, topical administration of NS2OS-H significantly decreased the corneal bacterial counts by 65%, whereas unmodified H and NS-H did not (Figure 5B). Together, these results indicate that sulfation at the C2 position of uronic acid is more important than sulfation at the C2 (amino group) and C6 positions of glucosamine for heparan compounds to inhibit *S. pneumoniae* adhesion onto FN fibrils and *S. pneumoniae* corneal infection. Furthermore, because both HP and 2-*O*-sulfated H can inhibit *S. pneumoniae* adhesion onto FN and corneal infection, the 2-*O*-sulfated uronic acid can be either GlcA or IdoA since HP is predominantly IdoA, whereas H only contains GlcA. In addition, while the conformation of IdoA can be either a ^4^C_1_ chair or ^2^S_0_ skew boat form, GlcA prefers a ^4^C_1_ conformation,^57^ suggesting that 2-*O*-sulfated uronic acids in the ^4^C_1_ conformation might play an important role in the inhibition of *S. pneumoniae* adhesion onto FN fibrils and corneal infection.

### Heparan compounds containing 2-*O*-sulfated motifs inhibit *S. pneumoniae* corneal infection in immunocompromised hosts and when administered therapeutically

*S. pneumoniae* consists of many serotypes, which differ in their tropism and infectivity of different tissues. Interestingly, an epidemiologic study at Massachusetts Eye and Ear Institute found that 95% of *S. pneumoniae* keratitis isolates are encapsulated, whereas 80% of conjunctivitis isolates are unencapsulated.^37^ While polysaccharide capsule may not be an essential virulence factor for *S. pneumoniae* keratitis^58,59^ the ongoing selective pressure of widely used vaccines targeting a small number of capsule serotypes has resulted in the emergence of novel serotypes not covered by these vaccines, which are becoming common colonizers of the nasopharynx and seem to be well adapted to cause keratitis.^37^

**FIGURE 6:**
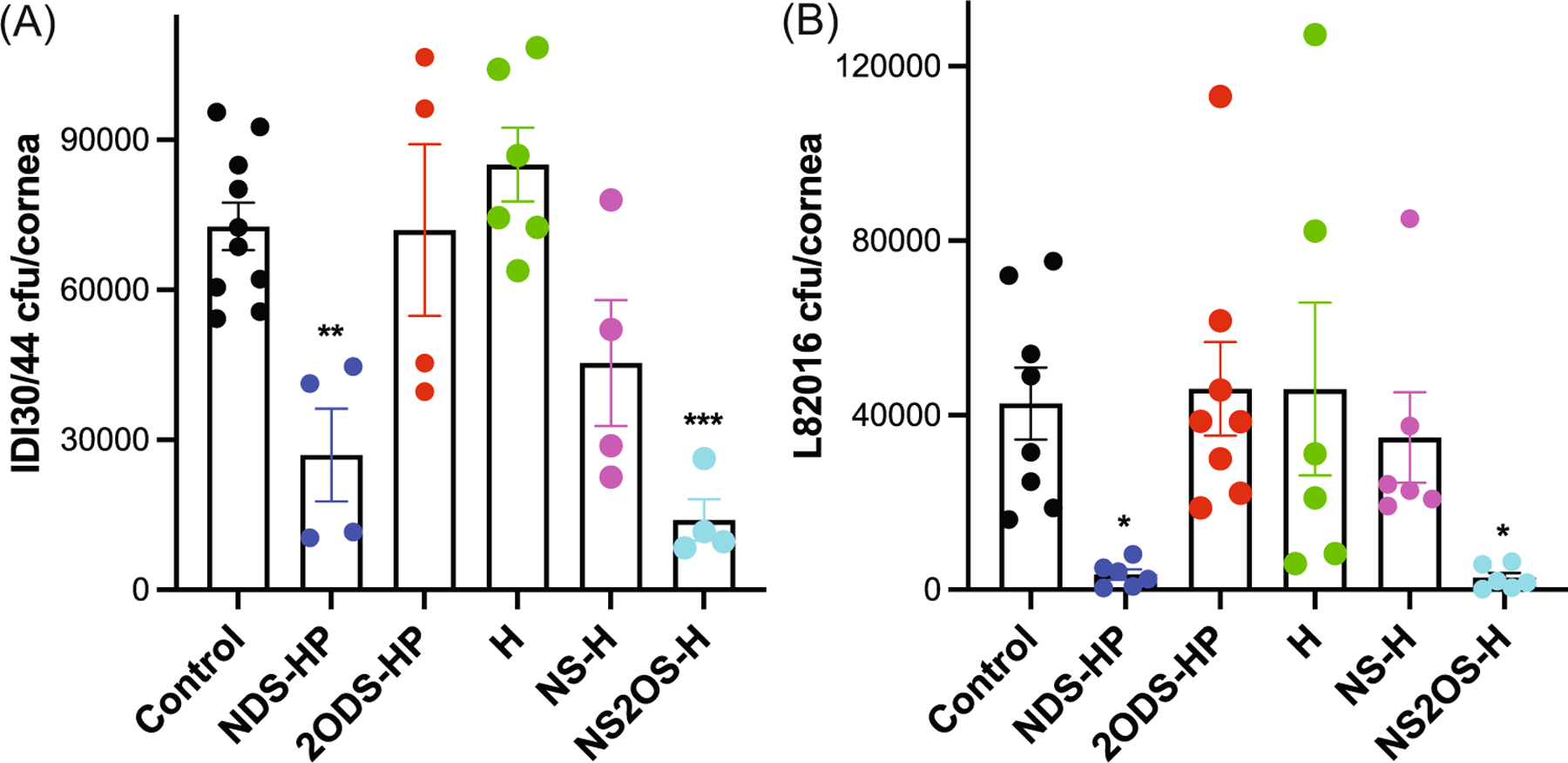
2-*O*-sulfated heparan compounds attenuate corneal infection by a clinical keratitis isolate of *Streptococcus pneumonia*. Injured mouse corneas were coinfected with (A) 4 × 10^8^ cfu of the clinical keratitis isolate IDI30/44 (mean ± SE, *n* = 4–10, ^**^p < 0.01, ^***^p < 0.001) or (B) 2× 10^8^ cfu of L82016 and PBS (control) or 300 ng of NDS-HP, 2ODS-HP, H, NS-H, or NS2OS-H, and the corneal bacterial burden was enumerated at 6 h pi (mean ± SE, *n* = 6–8, *p < 0.05). 2ODS-HP, 2-*O*-desulfated heparin; HP, heparin; NDS-HP, *N*-desulfated heparin; NS-H, *N*-sulfated H; NS2OS-H, *N*- and 2-*O*-sulfated H; pi, postinfection.

We, therefore, examined if 2-*O*-sulfated heparan compounds also inhibit corneal infection by other serotypes of *S. pneumoniae*, including the emerging serotype 35B. Injured mouse corneas were topically infected with IDI30/44 (serotype 35B), a clinical keratitis isolate, or with L82016 (serotype 6B) and NDS-HP, 2ODS-HP, H, NS-H, or NS2OS-H and the corneal bacterial burden was enumerated at 6 h pi. Similar to their effects on TIGR4 infection, only heparan compounds containing 2-*O*-sulfate groups significantly inhibited corneal infection by both serotypes 6B and 35B isolates. NDS-HP and NS2OS-H inhibited serotype 35B infection by 63% and 81%, respectively, whereas 2ODS-HP, H, and NS-H without 2-*O*-sulfates had no significant inhibitory effect on corneal bacterial counts (Figure 6A). Similarly, NDS-HP and NS2OS-H significantly inhibited serotype 6B corneal infection by 92% and 93%, whereas heparan compounds without 2-*O*-sulfate groups did not (Figure 6B). These data indicate that inhibition of *S. pneumoniae* corneal infection using heparan compounds could be potentially explored as a preventive approach in patients at higher risk or developing *S. pneumoniae* keratitis that is agnostic to the strain serotype.

**FIGURE 7:**
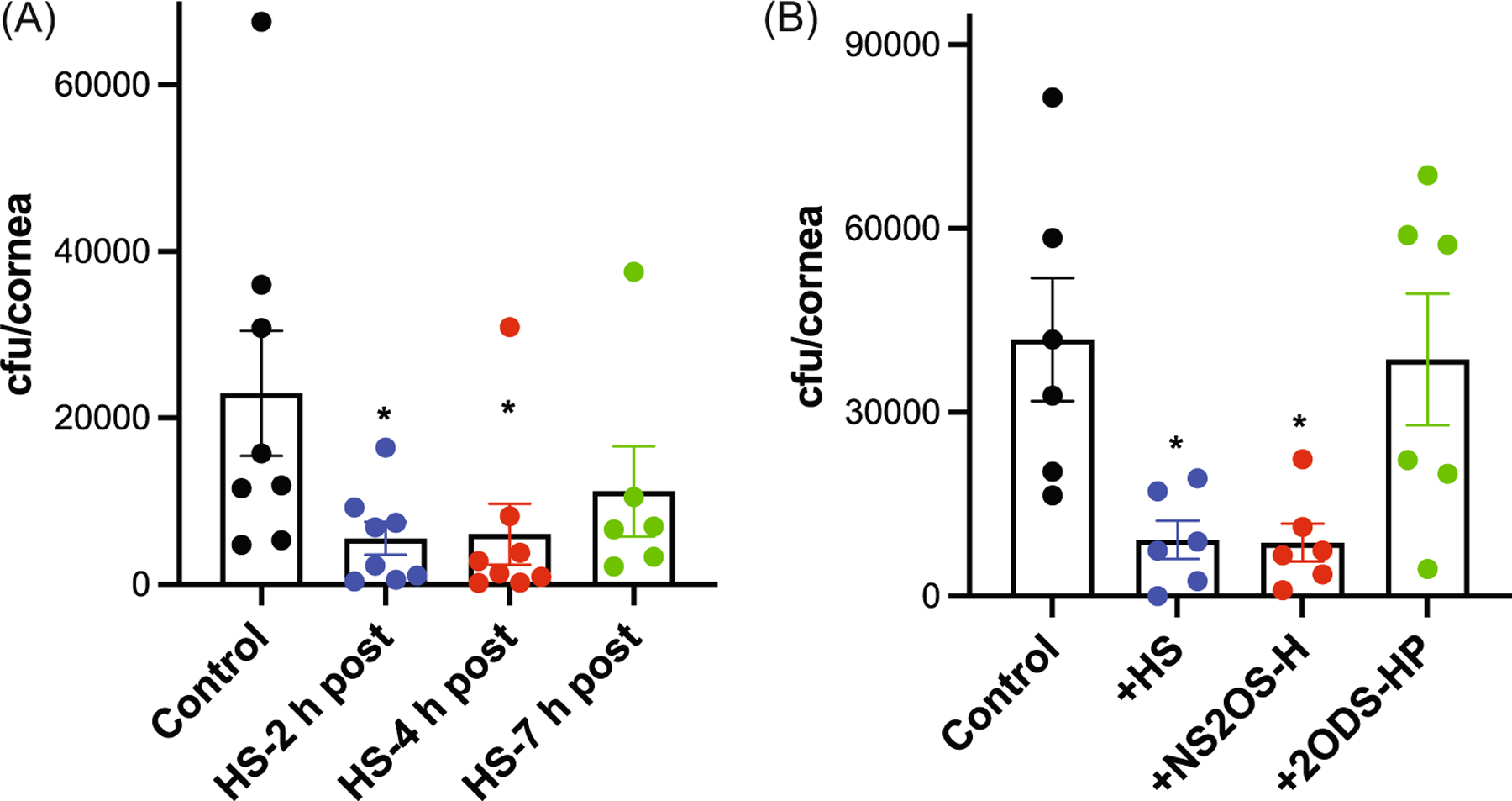
Sulfated heparan compounds inhibit *Streptococcus pneumoniae* corneal infection in immunocompromised hosts and when administered after infection. Mice were made neutropenic by injecting 100 μg of anti-Ly6G antibodies at 24 and 0.5 h before injury and infection. Injured corneas were infected with 3 × 10^8^ cfu of TIGR4 and topically administered: (A) 500 ng of HS at 2, 4, or 7 h pi or (B) 500 ng of HS, NS2OS-H, or 2ODS-HP at 2 h pi and the corneal bacterial burden was assessed at 12 h pi (mean ± SE, *n* = 6–8, *p < 0.05). 2ODS-HP, 2-*O*-desulfated heparin; HP, heparin; HS, heparan sulfate; NS2OS-H, *N*- and 2-*O*-sulfated H; pi, postinfection.

We next carried out experiments to examine the therapeutic value of heparan compounds. We first tested whether delayed administration of HS would protect against *S. pneumoniae* corneal infection in immunosuppressed hosts. Injured corneas of mice made neutropenic by injecting neutrophil-immunodepleting anti-Ly6G antibodies 1 day and 0.5 h before infection were topically infected with *S. pneumoniae* TIGR4 and treated with HS at 2, 4, or 7 h pi. The corneal bacterial burden was assessed at 12 h pi. HS administered at 2 and 4 h pi significantly decreased corneal bacterial counts by 76% and 74%, respectively (Figure 7A). HS administered at 7 h pi reduced the corneal bacterial burden by 51% but the reduction was not statistically significant. We also examined the effects of administering heparan compounds without or with 2-*O*-sulfates at 2 h pi and found that NS2OS-H significantly decreases corneal bacterial counts by 79% at 12 h pi, whereas 2ODS-HP did not (Figure 7B). These data suggest that bacterial adhesion to host tissue is a dynamic process that is important not only at the initial phase of pathogenesis but also during the progression of disease. Moreover, these results suggest that bacterial adhesion is a viable therapeutic target for *S. pneumoniae* keratitis.

## DISCUSSION

Our studies demonstrate that topical administration of HS, HP, and their structurally defined derivatives significantly inhibit *S. pneumoniae* corneal infection in mice. Analyses of *S. pneumoniae* adhesion onto the ECM deposited by cultured corneal epithelial cells by PLA revealed that *S. pneumoniae* binds directly to FN fibrils and that heparan compounds significantly inhibit this interaction in a manner similar to their effects on corneal infection. These data indicate that heparan compounds inhibit *S. pneumoniae* corneal infection by interfering with *S. pneumoniae* adhesion onto FN fibrils in the subepithelial ECM exposed by injury. Heparan compounds also inhibit corneal infection by several serotypes of *S. pneumoniae*, including a clinical keratitis isolate of an emerging serotype. Furthermore, heparan compounds inhibit *S. pneumoniae* corneal infection in immunosuppressed hosts and also when given pi, suggesting their therapeutic utility in *S. pneumoniae* keratitis.

Our studies with chemically modified HP compounds and chemoenzymatically sulfated H compounds suggested that inhibition of *S. pneumoniae* corneal infection and adhesion onto FN fibrils is not mediated by simple charge effects but by defined sulfate groups. Removal of *N*- or 6-*O*-sulfates from HP or addition of *N*-sulfates to H had no significant effect on the ability of HP or H to inhibit *S. pneumoniae* adhesion and infection. On the other hand, 2-*O*-desulfation significantly decreased the ability of HP to inhibit *S. pneumoniae* adhesion and infection, whereas the addition of *N*- and 2-*O*-sulfates enabled H to inhibit bacterial adhesion and infection, indicating that 2-*O*-sulfate groups drive the protective activities of heparan compounds. Furthermore, no significant inhibition was seen by HP oligosaccharides shorter than 6-mer, indicating that a length of at least 6-mer is required for significant inhibition of *S. pneumoniae* adhesion and corneal infection.

HS and HP can also inhibit other host–pathogen interactions where the pathogen uses HSPGs as receptors for their attachment. While many HS binding activities are thought to depend on the overall net charge of the glycosaminoglycan than on specific modifications,^60,61^ where examined, HS and HP inhibit host-pathogen interactions in a selective manner. For example, in *C. trachomatis* infection of epithelial cells, only HP oligosaccharides longer than dodecasaccharides and containing 6-*O*-sulfates inhibited infection.^62^ In hepatitis C virus infection of hepatoma cells, only HP oligosaccharides longer than decasaccharides and containing *N*- and 6-*O*-sulfate groups inhibited infection.^63^ The differences in size and sulfation requirements for inhibition illustrate the complex role of heparan compounds in modulating microbial pathogenesis.

Previous studies have shown that *S. pneumoniae* binds to the C-terminal region in immobilized FN,^64^ which contains the HP-binding HepII domain. HP inhibits *S. pneumoniae* binding to immobilized FN^64^ and our studies showed that HS and HP inhibit *S. pneumoniae* infection of injured corneas and *S. pneumoniae* adhesion onto FN fibrils in the corneal epithelial ECM. Together, these findings suggest that *S. pneumoniae* attaches to the HepII domain of FN in the corneal basement membrane. However, our studies also demonstrated that 2-*O*-sulfate groups are important in the ability of HS and HP to inhibit *S. pneumoniae* attachment to FN fibrils and infection of injured corneas. While several studies support the importance of 2-*O*-sulfates in FN binding by HS and HP, there are also studies that indicate otherwise. For example, a study that examined binding of lung HP to HepII suggested that while all sulfate positions may contribute to binding, the hierarchy of importance for binding was determined to be in the order of 2-*O*-sulfates » 6-*O*-sulfates > *N*-sulfates.^65^ Loss of *N*-sulfates or neutralization of carboxyl groups had minimal effects on lung HP binding to HepII, but complete loss of 2-*O*-sulfates increased the IC_50_ by 20-fold,^65^ demonstrating that 2-*O*-sulfates are required for high-affinity binding of lung HP to HepII. Similarly, molecular docking studies of HepII domain with a series of differentially sulfated HP dodecasaccharides have shown a hierarchical importance of 2-*O*-sulfates » 6-*O*-sulfates > *N*-sulfates in HepII binding.^66^ Furthermore, studies with HP mimetics have demonstrated that both 2-*O*- and 6-*O*-sulfated mimetics and *N*-, 2-*O*-, and 6-*O*-sulfated mimetics promote FN fibrillogenesis by CHO-677 cells as well as intact HP, suggesting that 2-*O*- and 6-*O*-sulfates are important, but *N*-sulfates are dispensable for FN binding and subsequent facilitation of FN assembly.^67^ On the other hand, removal of *N*-sulfates was found to inhibit the ability of mucosal HS to bind to HepII, whereas selective loss of over half of the 6-*O*-sulfates or most of 2-*O*-sulfates had minimal inhibitory effects,^65^ suggesting that *N*-sulfates, and not 2-*O*- or 6-*O*-sulfates, are critical for HepII binding by mucosal HS. HS isolated from wild type and *Hs2st*^−/−^ embryonic cells also showed a similar binding affinity to FN.^68^ Furthermore, in a study examining the inhibitory effects of HS on fibroblast adhesion to HepII, HP oligosaccharides longer than 14mer were required for optimal inhibition, and *N*-sulfate groups were found to be essential, but the impact of 2-*O*-sulfate and 6-*O*-sulfate groups on HepII binding was determined to be marginal.^69^

Broadly speaking, these data suggest that 2-*O*-sulfate groups are required for HP binding to HepII and FN, whereas *N*-sulfates are required for HS binding to HepII, which is different from the similar inhibitory effects of HS and HP observed in our study. Clearly, differences in experimental systems may have contributed to the discrepant observations. For example, we measured the inhibitory effects of HS and HP on *S. pneumoniae* adhesion onto FN fibrils assembled by corneal epithelial cells, but most studies used human plasma FN and measured HS/HP binding to FN or HepII coated on plastic surfaces. Alternatively, FN-binding proteins of *S. pneumoniae* might interact with regions in the vicinity, but not directly to the HepII domain. In fact, although *S. pneumoniae* binds to the C-terminal FN fragment and this binding is inhibited by excess HP, inhibition by excess 40 kDa HepII fragments is incomplete.^64^ Hence, *S. pneumoniae* may interact with other FN domains in the C-terminal end where binding is susceptible to inhibition by HP and HS. Furthermore, *S. pneumoniae* shows enormous plasticity in its ability to interact with FN as it expresses several FN-binding proteins on its surface, including PavA, PavB, endopeptidase O, RrgA, PfbA, and PfbB.^17,70,71^ Although PavA is thought to be the dominant FN-binding protein that mediates *S. pneumoniae* binding to FN fibrils during corneal infection,^12^ the role of other FN-binding proteins in corneal pathogenesis has not been tested. In addition, PavA has been shown to bind to various synthetic peptides corresponding to amino acid sequences in FN type III repeats,^17^ where all of these type III repeat peptides are highly cationic with pIs greater than 11, suggesting that anionic HS and HP may bind to these regions and impede bacterial binding. Together, these observations point to the possibility that *S. pneumoniae* might bind to several FN domains in the C-terminus when infecting injured corneas, and that heparan compounds inhibit these interactions in a 2-*O*-sulfate-dependent manner.

Our studies demonstrate that inhibition of corneal infection in vivo by 2-*O*-sulfated heparan compounds is more prominent compared to inhibition of bacterial adhesion onto the corneal epithelial ECM in vitro. 2-*O*-sulfated heparan compounds almost completely inhibited *S. pneumoniae* infection of injured corneas, but only inhibited *S. pneumoniae* adhesion by approximately 60%. While the inhibitory effects were significant, the incomplete inhibition of bacterial adhesion suggests that the ECM assembled by corneal epithelial cells might contain additional adhesive ligands for *S. pneumoniae* that are not susceptible to inhibition by 2-*O*-sulfated heparan compounds. The corneal epithelial ECM also contains large amounts of basement membrane components, such as laminin, perlecan, and nidogen. Although PLA showed that *S. pneumoniae* does not bind to laminin and the low level of laminin binding is not inhibited by HS, the potential binding of *S. pneumoniae* to other basement membrane components has yet to be directly examined. Alternatively, a 60% inhibition of bacterial adhesion may simply be sufficient to allow host defense mechanisms to effectively eradicate *S. pneumoniae* in vivo.

In sum, our studies clearly demonstrate that 2-*O*-sulfated heparan compounds significantly prevent and can also treat *S. pneumoniae* corneal infection. Whether 2-*O*-sulfated heparan compounds inhibit corneal infection by other ocular surface pathogens is not known, but other major causative agents of bacterial keratitis also bind to FN. For example, *P. aeruginosa* binds to FN via OprQ^72^ and *S. epidermidis binds* to FN via Embp.^18,19^ The site in FN that *P. aeruginosa* binds to is not known, but *S. epidermidis* binds to the HepII domain of FN, suggesting that, in principle, HS and HP may also inhibit *S. epidermidis* keratitis. Thus, the potential therapeutic impact of inhibiting FN binding in bacterial keratitis by heparan compounds may extend beyond *S. pneumoniae* infection. Moreover, *S. pneumoniae* is also the leading cause of community-acquired pneumonia and one of the major causes of otitis media, sepsis, and meningitis.^73^ While the importance of FN binding in these pneumococcal diseases has not been clearly established, deletion of the PavA gene significantly reduces *S. pneumoniae* virulence in mouse models of sepsis,^74^ pneumonia,^2^ and meningitis.^75^ These results suggest that examining the inhibitory effects of 2-*O*-sulfated heparan compounds on *S. pneumoniae* adhesion and infection of other tissues may be well worth trying.

## Supplementary Material

Fig.S3

Fig.S4

Fig.S2

Fig.S1

Table S1

## Data Availability

The data that support the findings of this study are available from the corresponding author upon reasonable request.

## Abbreviations:

2ODS-HP: 2-*O*-desulfated heparin
6ODS-HP: 6-*O*-desulfated heparin
ECM: extracellular matrix
FN: fibronectin
GlcA: glucuronic acid
GlcNAc: *N*-acetylglucosamine
GlcNS: *N*-sulfated glucosamine
H: heparosan
HP: heparin
HS: heparan sulfate
HSPGs: heparan sulfate proteoglycans
IdoA: iduronic acid
NDS-HP: *N*-desulfated heparin
NS2OS-H: *N*- and 2-*O*-sulfated heparosan
NS-H: *N*-sulfated heparosan
PAPS: 3′-phosphoadenosine 5′-phosphosulfate
PLA: proximity ligation assay
PNAG: poly-*N*-acetylglucosamine
S. pneumoniae: Streptococcus pneumoniae
TSA: trypticase soy agar
αSMA: alpha smooth muscle cell actin
