# Supplementary figures and images for: Sulfated motifs in heparan sulfate inhibit Streptococcus pneumoniae adhesion onto fibronectin and attenuate corneal infection
Source: Proteoglycan Res. Author manuscript; Available in PMC 2024 Jul 2. (PMC11218895; doi:10.1002/pgr2.9)

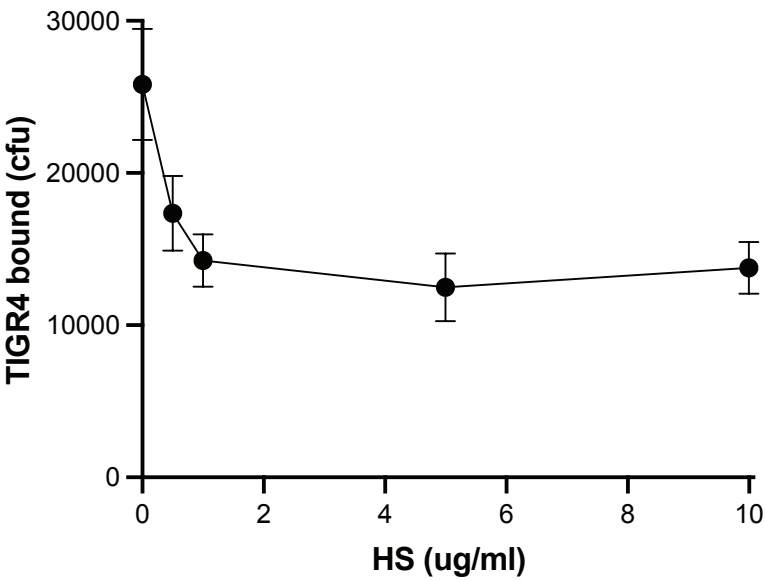

Supplement: Fig.S3 [file NIHMS1950838-supplement-Fig_S3.pdf]

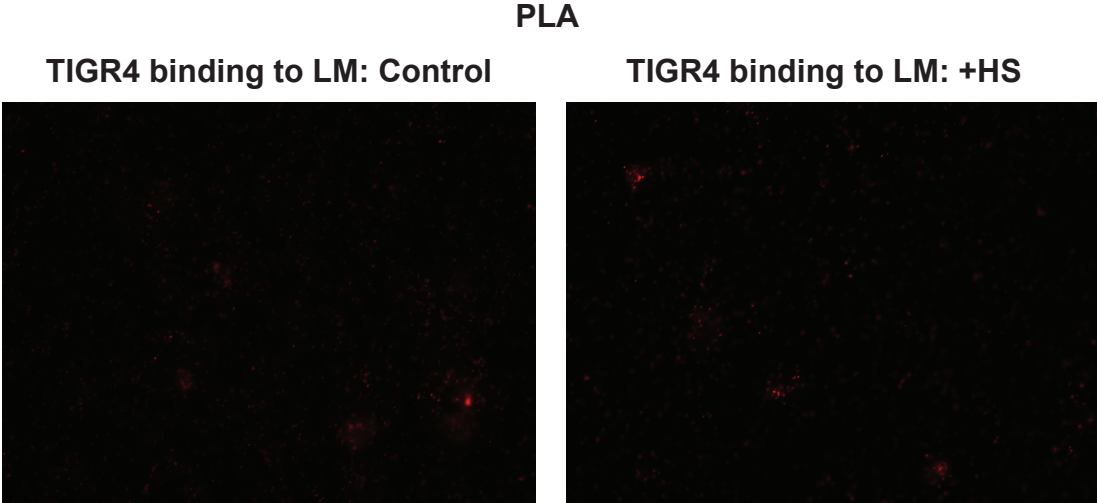

Supplement: Fig.S4 [file NIHMS1950838-supplement-Fig_S4.pdf]

Immunostaining of A6(1)  
decellularized ECM

Laminin

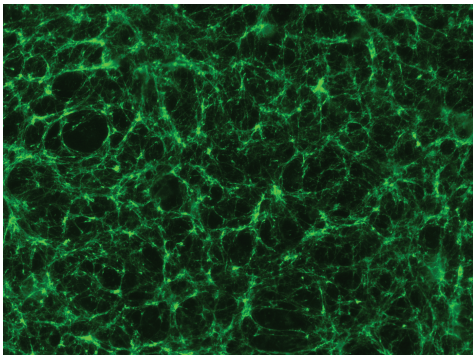

Nidogen

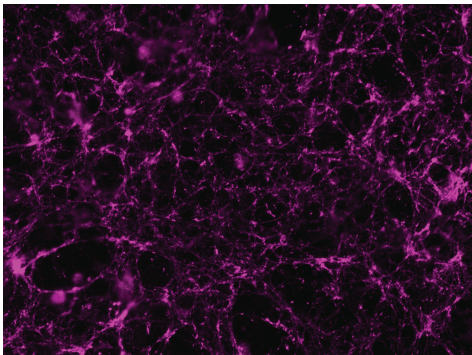

Supplement: Fig.S2 [file NIHMS1950838-supplement-Fig_S2.pdf]

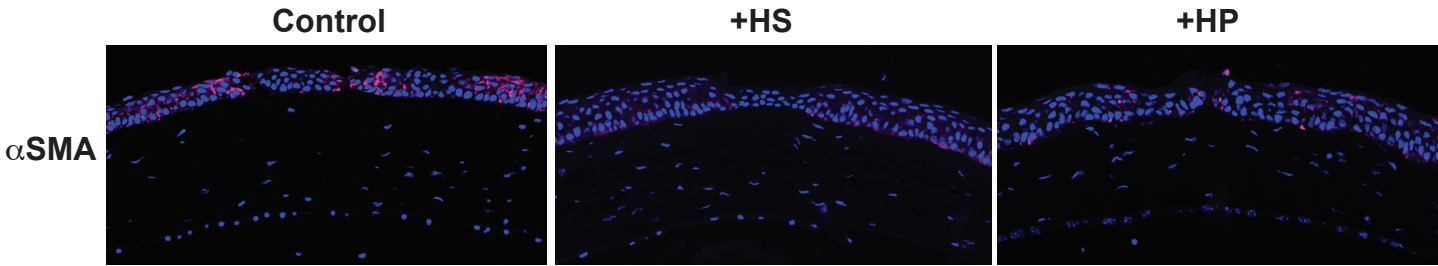

Supplement: Fig.S1 [file NIHMS1950838-supplement-Fig_S1.pdf]
