# Supplementary material for: Sulfated motifs in heparan sulfate inhibit Streptococcus pneumoniae adhesion onto fibronectin and attenuate corneal infection
Source: Proteoglycan Res. Author manuscript; Available in PMC 2024 Jul 2. (PMC11218895; doi:10.1002/pgr2.9)
Supplement: Table S1 [file NIHMS1950838-supplement-Table_S1.docx]

**Table S1. Disaccharide composition of desulfated heparin**

| Sample | | HS disaccharides (%) | | | | | | | |
| --- | --- | --- | --- | --- | --- | --- | --- | --- | --- |
|  |  | TriS | NS6S | NS2S | NS | 2S6S | 6S | 2S | 0S |
| 2ODS-HP | 1 | 0.56 | 65.72 | 0.26 | 20.59 | 0.00 | 4.61 | 0.00 | 8.26 |
|  | 2 | 0.62 | 63.43 | 0.16 | 20.87 | 0.36 | 4.46 | 0.00 | 10.10 |
|  | average | 0.59 | 64.58 | 0.21 | 20.73 | 0.18 | 4.53 | 0.00 | 9.18 |
| 6ODS-HP | 1 | 6.03 | 7.56 | 30.06 | 22.29 | 0.00 | 3.98 | 2.10 | 27.98 |
|  | 2 | 5.54 | 7.31 | 30.60 | 23.38 | 0.00 | 2.72 | 3.20 | 27.24 |
|  | average | 5.78 | 7.44 | 30.33 | 22.84 | 0.00 | 3.35 | 2.65 | 27.61 |
| NDS-HP | 1 | 0.05 | 0.00 | 0.00 | 0.00 | 16.07 | 35.21 | 3.38 | 45.30 |
|  | 2 | 0.01 | 0.00 | 0.00 | 0.00 | 14.02 | 35.02 | 5.04 | 45.91 |
|  | average | 0.03 | 0.00 | 0.00 | 0.00 | 15.05 | 35.11 | 4.21 | 45.60 |
| Parent  HP | 1 | 47.68 | 11.43 | 6.31 | 5.07 | 4.23 | 7.22 | 1.31 | 16.77 |
|  | 2 | 46.60 | 11.90 | 6.06 | 6.17 | 3.78 | 7.74 | 1.66 | 16.10 |
|  | average | 47.14 | 11.67 | 6.18 | 5.62 | 4.00 | 7.48 | 1.48 | 16.43 |
